# A qualitative exploration of perceived needs and barriers of individuals with schizophrenia, caregivers and clinicians in using mental health applications in Madhya Pradesh, India

**DOI:** 10.1016/j.ssmmh.2022.100063

**Published:** 2022-12

**Authors:** Ameya P. Bondre, Ritu Shrivastava, Harikeerthan Raghuram, Deepak Tugnawat, Azaz Khan, Snehil Gupta, Mohit Kumar, Urvakhsh Meherwan Mehta, Matcheri Keshavan, Tanvi Lakhtakia, Prabhat Kumar Chand, Jagadisha Thirthalli, Vikram Patel, John Torous, Abhijit R. Rozatkar, John A. Naslund, Anant Bhan

**Affiliations:** a Sangath, 120 Deepak Society, Chuna Bhatti, Bhopal, Madhya Pradesh, 462016, India; b All India Institute of Medical Sciences (AIIMS), Saket Nagar, Bagh Swaniya, Bhopal, Madhya Pradesh, 462020, India; c National Institute of Mental Health and Neurosciences, Hosur Road, Lakkasandra, Wilson Garden, Bengaluru, Karnataka, 560029, India; d Division of Digital Psychiatry, Beth Israel Deaconess Medical Center (BIDMC), 330 Brookline Ave, Boston, MA, 02215, United States; e Harvard Medical School, 25 Shattuck St, Boston, MA, 02115, United States

**Keywords:** Digital health, mHealth, Qualitative, Mental health, Schizophrenia, Smartphone application, Global mental health, LMIC, Low-resource setting

## Abstract

**Introduction:**

About 3.5 million people are living with schizophrenia in India, with most failing to receive minimally adequate care. Digital mental health applications could potentially decrease this treatment gap; however, these applications should be tailored to meet the needs and overcoming barriers of its end-users to ensure their adoption and sustained usage. Few studies in India have explored the perspectives of target stakeholders to understand how digital tools could be viable for supporting care. Therefore, this study explores the perceived needs and barriers of patients with schizophrenia, caregivers and clinicians in using digital mental health applications.

**Methods:**

Focus group discussions (FGDs) were conducted with patients having schizophrenia attending outpatient clinics at a government tertiary hospital, and their caregivers, and mental health clinicians in Bhopal, Madhya Pradesh, India. FGDs were audio-recorded and coded. Framework analysis was employed to guide the analysis, involving deductive and inductive generation of themes, data triangulation and comparison of perspectives between participant groups.

**Results:**

Six FGDs were conducted with individuals with schizophrenia (n ​= ​11), their caregivers (n ​= ​14), and mental health clinicians (n ​= ​19). Four *a priori* themes were established: a) Prior experiences with health applications; b) Content of a mental health application; c) Involvement of caregivers in mental health application usage and d) Supporting doctors’ work through mental health applications. Additionally, two themes were generated inductively: a) Qualities of a mental health application and b) Data privacy and confidentiality.

**Conclusion:**

Exploration of stakeholder perspectives on the content, features, and uses of mental health applications is crucial to yield initial insights about the use of these digital programs in India. This study generated a multitude of suggestions on app functionality and components, which can guide ongoing efforts to develop and deliver digital mental health applications for patients living with schizophrenia in low-resource settings, with limited access to mental health services.

## Highlights

• Patients preferred an app that probes symptoms and gathers urgent concerns.
• Clinicians wanted to know more about target symptoms and details of patients’ mood.
• Both groups advised to capture users’ daily activities to assess mental balance.
• Clinicians found app utility in their daily tasks e.g., logging patient history.
• Patients generally agreed for their data sharing e.g., for customized suggestions.

## 1 Introduction

Schizophrenia affects 20 million people globally (GBD 2017 Disease and Inju, 2018) and exerts a substantial burden on patients and their caregivers in terms of illness cost and disability (Kennedy et al., 2014; Kleine-Budde et al., 2014; Somaiya et al., 2014). There are approximately 3·5 million people living with schizophrenia in India (Sagar et al., 2020). Psychosocial interventions, in addition to medications, are critical to enable patients living with schizophrenia to improve their independence, functioning and productivity (Mueser et al., 2013). However, access to psychosocial interventions for patients and their caregivers is severely limited in a country like India given the scarcity of mental health professionals; there is approximately one psychiatrist for every ∼333,000 people (Patel et al., 2016), with a concentration of specialized mental health personnel in the cities (Lahariya, 2018), a scarcity of integration of mental health care into routine primary care (Balamurugan et al., 2019; Rathod et al., 2017), and a dire shortage of mental health resources (Patel et al., 2016). Over 80% of patients suffering from severe mental illnesses, such as schizophrenia, fail to receive minimally adequate treatments (Lund et al., 2012; Srivastava et al., 2016), creating an alarming mental health ‘treatment gap’. Therefore, there is an urgent need for innovative interventions to support care of individuals living with schizophrenia, and scaling up community-based services in conjunction with existing clinical treatments.

With increasing access to digital technologies, including smartphones and greater mobile internet connectivity across the country, digital solutions could potentially yield novel approaches for decreasing the treatment gap for schizophrenia (Aggarwal, 2012; Rodriguez-Villa et al., 2021a). For instance, this could be achieved by reducing stigma, as patients may feel more comfortable in reporting their concerns via personalized calls, messages or in-app communication, compared to clinic appointments (Aggarwal, 2012); allowing patients to access peer support by individual or group calls, and seeking remote support from lay providers, which maybe preferred by patients over meeting with physicians or psychiatrists (Kermode et al., 2009); and increasing service access by bringing interventions to the patient rather than requiring the patient to go to the health facility, as could be supported through the relay of information to the patient on treatment adherence, appointment reminders, and physician queries via text messaging (Aggarwal, 2012; Mahmud et al., 2010). Smartphone-based psychosocial interventions for persons living with schizophrenia, therefore, may focus on mental health literacy, medication adherence, prevention and management of relapse or worsening of symptoms, self-monitoring/self-care, vocational support, and psychosocial support for family members.

There is a paucity of studies describing digital mental health applications for schizophrenia in resource-limited settings such as India (Carter et al., 2021; Merchant et al., 2020). Existing evidence points to challenges in the development of smartphone applications, and in sustaining usage among patients and caregivers (Larsen et al., 2019; Naslund et al., 2015; Nicholas et al., 2015; Toefy et al., 2016). Many commercial smartphone applications are not often rigorously tested and, in some cases, could potentially increase risk of harm for users (Naslund et al., 2015). Studies have found such applications to have content that does not align with established practice guidelines or self-management principles (Nicholas et al., 2015), or does not adequately involve individuals with lived experience of mental illness in the development process (Larsen et al., 2019). From a mental health user-perspective, studies have shown that since most of the available applications have commercial purposes, their development processes focus more on the novelty of app operations and features, rather than acceptability and usability of technology among users (Toefy et al., 2016), which reduces long-term app usage. In addition, subscription-based or paid applications significantly limit access among those in low- and middle-income countries.

Furthermore, users’ engagement with the application may be mediated by their perceived and actual challenges of using technology, which is even true for simple SMS-based reminders for adhering to medications, as patients may perceive those as an irritation or disturbance (Kannisto et al., 2015). There are also potential threats to the utility of the application design when users are not closely involved in the application development process (Ben-Zeev et al., 2013), as also by an imbalance of technology expertise between developers and users, reflected by low digital literacy of patients and caregivers, a concern that is particularly pronounced in low-resource settings (McCurdie et al., 2012). Many individuals living with schizophrenia also experience potential cognitive or motor functioning challenges that can hamper application usage (Merchant et al., 2020), further reinforcing the need to rigorously co-develop applications with patients, caregivers and clinicians to overcome these limitations. On the whole, it is necessary for intervention developers to consider user behavior in smartphone usage, technological literacy and clinical factors to develop a tailored digital intervention, which reinforces the need for collaboration with local participants who would be the end-users of the intervention (Hahn & Inhorn, 2009).

Few studies in India have closely involved target users across the development, piloting and deployment of a digital mental health intervention (Sinha et al., 2018). It is essential to study the needs, perspectives and challenges of patients, caregivers and clinicians, to better understand how these potential digital tools could be viable for supporting care, and to inform their effective design. The use of ‘participatory methods’ (Chancellor et al., 2019) can enable various users to share insights through a series of focus group discussions. Moreover, there is a need to build more evidence on participatory qualitative studies in India in government health settings, as they serve a wide range of patients from various socio-economic backgrounds, particularly in regions with high treatment gaps in mental health.

Therefore, this qualitative study seeks to explore the perceived needs and barriers of patients with schizophrenia, their caregivers and mental health professionals in using digital mental health applications. These findings will inform the design, adaptation and roll out of a digital mental health application (section 2.1) for management of schizophrenia in low-resource settings.

## 2 Material and methods

### 2.1 Study setting

This qualitative study is nested within a larger project called SHARP (Smartphone Health Assessment for Relapse Prevention (Rodriguez-Villa et al., 2021a)) focused on the systematic development and adaptation of an open-source smartphone application called “mind-LAMP” (Learn, Assess, Manage, Prevent) across different cultures and contexts to prevent relapse among individuals diagnosed with schizophrenia spectrum disorders. This study was conducted at the All India Institute of Medical Sciences (AIIMS), a government funded tertiary care hospital in Bhopal, Madhya Pradesh, between October 2020 and March 2021. AIIMS Bhopal represents a leading regional institution for provision of psychiatric care, and specifically, treatment of schizophrenia and severe mental illnesses. Madhya Pradesh is a state where upwards of 90% of people living with mental illness do not have access to care (Kokane et al., 2019) and the ‘contact coverage’ (proportion of people with mental illnesses who seek care for their symptoms) is as low as 13% (Shidhaye et al., 2017). Ethical approval was obtained from each institution involved in this project, including AIIMS Bhopal, India; the National Institute of Mental Health and Neurosciences in Bengaluru, India; Sangath, India; and Beth Israel Deaconess Medical Center in Boston, USA. A joint application was approved by the Health Ministry Screening Committee at the Indian Council of Medical Research, New Delhi.

### 2.2 Study procedures

We conducted a secondary analysis of data obtained from the parent SHARP project (Rodriguez-Villa et al., 2021a). Perspectives of patients, caregivers and clinicians on the use of digital mental health interventions were collected through focus group discussions. Specifically, our aim was to gather participants’ insights about mental health applications, and the features, visualisations and functionality aspects that they perceive as important, which make an application most compatible for daily usage.

We used a flexible, semi-structured topic guide based on a review of relevant literature, and informed by prior guidelines on focus group discussions (FGDs) (Acocella, 2012; Krueger, 2014; Saunders et al., 2008). The topic guide broadly explored the experiences of participants in routine smartphone usage and their reasons to continue or discontinue using applications of general use; the perspectives on health applications including the barriers and expectations out of mental health applications, aspects of data privacy and confidentiality, and role of caregivers in mental health applications; as well as the perspectives of clinicians on mental health applications that can facilitate routine clinical work, documentation of patient treatment and communicating with patients and families.

We extracted data from transcripts pertaining to participant perspectives towards mental health applications. We recognize that focus groups with a diverse group of participants and resulting group dynamics can reflect conversations on specific topics in a natural setting, and thus, reduce moderator bias, compared to interviews, where participants’ views could be influenced by the interviewer (Krueger, 2014). We conducted two separate types of focus group discussions: those involving mental health professional cadres or ‘clinician FGDs’; and involving both patients and caregivers or ‘patient-caregiver FGDs’. A mixed group helped us in initiating discussions, encouraging diverse points of view and cross-communication between various mental health cadres, and between caregivers and patients, and promoted a wider perspective to the problems faced by patients in their routine life, clinicians in delivering services, and caregivers in providing support to patients. The FGDs, averaging 60 ​min in duration, were conducted by experienced facilitators (RS, HR and APB) as part of an iterative and inductive data collection process.

The FGD was preceded by a brief orientation to the parent study procedures followed by administration of the consent process – a written informed consent was obtained at the beginning of each person’s participation in the study. Inclusion in the study did not have any bearing on the patient’s outpatient treatment or hospital stay, and similarly had no impact on the clinicians’ role within their clinical setting. All participants had the right to decline any information or leave the study at any point, without consequence. Our team was also sensitive to the fact that individuals living with schizophrenia represent a vulnerable group, which is more likely to experience social and cultural disadvantages relative to the general population. Therefore, to ensure their comfort and safety, FGDs were conducted by trained researchers, and supportive referral protocols were put in place in response to any potential psychiatric emergency (e.g., feelings of distress).

After completion of the consent procedure, the FGDs were moderated as per the topic guide with consistent support offered by the moderators to the participants, for the latter to freely express their views and discuss as a group. The order in which the topics emerged in the discussion was flexible – it was influenced but not exclusively mediated by the guide. Each FGD would typically include 7–8 participants and two to three moderators and was audio-recorded using two voice-recorders. An additional observer (or the non-participating moderator) collected notes on participants’ responses, non-verbal cues and overall behaviour observations during the FGDs, and these were used to supplement the data analysis.

### 2.3 Participant recruitment

Adult patients (at least 18 years of age) diagnosed with schizophrenia (as per the International Statistical Classification of Diseases and Related Health Problems 10th Revision (ICD-10) (World Health Organization, 1992)), either attending outpatient clinics at the Department of Psychiatry, AIIMS Bhopal, or clinically stable ambulatory in-patients were planned to be included. Primary caregivers of these patients, who were themselves not suffering from any mental or cognitive disorder, were invited to participate. Mental health professionals, such as psychiatrists (including residents), clinical psychologists, psychiatric social workers, or psychiatric nursing professionals, working at AIIMS-Bhopal or other hospitals and clinics in Bhopal, were also invited to participate in the study. We used a non-probabilistic sampling approach to recruit a maximum-variation purposive sample (Bucci et al., 2019) of clinical staff in the Department of Psychiatry at AIIMS-Bhopal and other health facilities in Bhopal, and patients and caregivers attending outpatient clinics at AIIMS-Bhopal.

### 2.4 Data collection and processing

All FGDs were conducted in Hindi or English, audio-recorded and subsequently transcribed verbatim and translated into English. All audio-files were accessed only by the researchers who conducted the FGDs. As part of initial review, three researchers (RS, HR, APB) independently reviewed raw audio files for clarity and quality. Thereafter, these files were independently transcribed and Hindi quotes were translated into English for consistency of language. Any queries on participants’ responses or audio clarity issues were discussed between the researchers and resolved, to maintain coherence of the transcript content. The complete transcript was reviewed, before initiation of preliminary ‘open-text categorizing’, to facilitate the subsequent process of coding and thematic analysis.

### 2.5 Data analysis

Data were analysed using a framework analysis approach (Ritchie et al., 2003). Framework analysis flexibly allows the inclusion of both *a priori* themes, or ‘deductive’, pre-existing themes deduced from the FGD guide, and themes emergent in the course of the analysis or ‘inductive’ themes. In collaboration with senior researchers (JN, JT, AB), the on-site researchers at Sangath (RS, HR and APB) developed the initial coding framework, reflecting important areas of exploration, before reviewing the transcripts and further developing the framework. The topic guide essentially informed the framework’s *a priori* themes. After independently coding the transcripts, we refined the framework using inductive themes, and after consultation with the wider research team, we allowed subsequent iterations in the framework.

Development of the framework involved nonlinear stages. This started with familiarization with the data, where we listened to recordings, made notes and became immersed in the transcribed content. Next, we coded the data, arriving at the aforementioned deductive and inductive themes. Thereafter, we developed and refined our thematic coding framework, where the initial framework was developed through deductive FGD guide-based themes and the independent coding of transcripts assigned emergent/inductive themes to the framework. During this process, the pre-existing and new themes were compared and a set of themes was agreed upon to finalize the framework. This process also involved interpretation and summarization of data, addition of new themes, deletion of redundant themes, and integration of overlapping themes. Further, in a process referred to as ‘indexing’, we identified sections of the transcript textual data that corresponded to particular themes. We extracted this original textual data and placed it in charts with headings and subheadings, drawn from the thematic coding framework. We used QSR International’s NVivo 12 data management software to facilitate this process (QSR International, 1999). Lastly, in a process of mapping and analysis, we identified connections between themes, and explored relationships (similarities and differences) between concepts.

RS, HR and APB held regular consensus meetings towards developing a framework, which was reviewed by senior researchers in the team (JT, JN, AB, DT). The framework was not completed or finalized until all the transcripts were coded and analysis of information obtained from clinicians, patients and caregivers was triangulated (Ritchie et al., 2003). Therefore, the framework allowed for a comparison of perspectives from patients, caregivers and clinicians, to account for the possibility of overlapping (or contrasting) themes between different groups of participants. During the analysis, we have stratified the themes as per the participant group wherever possible (i.e., patients-caregivers, and clinicians), to clarify these differences and similarities.

## 3 Results

### 3.1 Study sample

We conducted 3 FGDs with 11 patients and 14 caregivers, and 3 FDGs with 19 clinicians. The final clinician FGD was conducted remotely over Zoom videoconferencing software due to restrictions on in-person gatherings imposed by the COVID-19 pandemic. Participant demographic characteristics are summarized in Table-A.1.

### 3.2 Deductive ‘*a priori*’ themes

**Fig. A.1:**
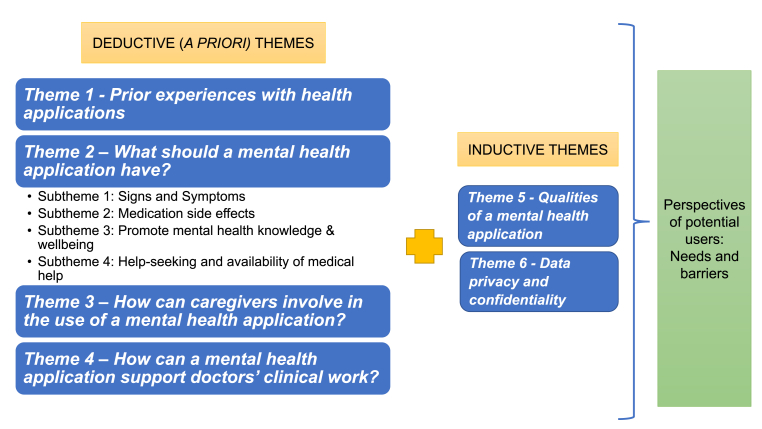
Themes and Subthemes for Analysis. Note: Deductive themes were based on the FGD guide and inductive themes emerged in the course of data analysis. Taken together, they provided perspectives on needs and barriers in using mental health applications among patients, caregivers and clinicians.

A key part of our thematic analysis involved exploring four themes that were established *a priori* (Figure A.1). These include: prior experiences with health applications; perceived content of a mental health application; involvement of caregivers in the use of a mental health application and, use of a mental health application to support clinical work. We summarize these themes in the sections that follow, and include representative quotes in Table A 2.

#### 3.2.1 Theme 1 – Prior experiences with health applications

We explored participants’ prior exposure to digital health applications to understand their views on using these applications and whether these apps were helpful or not. Patients and caregivers commented on their usage of lifestyle modification applications, with perceived benefits on physical health, and possibility to derive mental health gains from physical fitness applications. Participants also mentioned the utility of applications with details on available medications, and highlighted that a single application providing comprehensive and relevant information would be especially helpful. Participants also cited interest in applications to learn more about their own health condition, or to obtain medical help from a qualified expert.

Among mental health clinicians, they mentioned having recommended lifestyle modification applications to their patients, where simple features of a diet application and contextually relevant food items were underscored. In another instance, a clinician discussed the presence of coaching for lifestyle modification, for respondents who may have little awareness. One clinician pointed out the need for piloting lifestyle modification applications among rural, low-literacy patients, while recognizing that these patients would need support of family members in understanding app usage, and ensuring access to a smartphone. Clinicians also recognized the utility of apps that could enable online placement of medication requests and dispatch of medicines. Additionally, clinicians highlighted key app characteristics such as easy language, use of simple, practical terminology, and means of establishing a connection or contact with the health expert/doctor.

Given the pandemic, patients/caregivers and clinicians also observed the need of mental health apps to facilitate access to treatment when in person meetings were not possible, or to ensure availability of medicines/counselling for patients.

#### 3.2.2 Theme 2 – What should a mental health application have?

We were interested in eliciting participants’ needs and expectations out of digital mental health applications. Despite participants’ limited exposure to these types of applications, they offered important suggestions for developing or improving the features and content of mental health applications. Specifically, participants mentioned key topics related to signs and symptoms, medication side-effects, promotion of mental health knowledge and wellbeing, help seeking and availability of medical help. The findings are illustrated in the sections below, and summarized in Table A 2.

##### 3.2.2.1 Sub-theme – signs and symptoms

Several participants described use of an application for self-assessment of signs and symptoms, or reporting of mental health concerns, to seek answers or solutions to help with their mental health concerns. Patients and caregivers emphasized the importance of queries on symptoms or symptom relief and precautions. They expressed preference for self-assessment questionnaires that could generate more clarity for addressing any prevailing symptoms or difficulties, or probe into emerging symptoms. Some participants were open to having a system to raise an alarm or communicate urgent symptoms, depicted as a ‘panic button’ for connecting with doctors, and even other social/family contacts, to ensure early intervention. Participants also expressed interest in features for specific symptoms, such as addressing anxiety.

Clinicians recognized the need for applications to improve the understanding of patient users about the symptoms of mental disorders or focus on ‘psychoeducation’. Clinicians expressed a need to obtain information on patient’s target symptoms and early warning signs of relapse, and have means to achieve immediate consultation and scope for patient’s self-assessment. Clinicians also commented that a mental health app should prioritise identifying ‘target symptoms’, such as common observed symptoms in schizophrenia. Clinicians also stressed the inclusion of early warning or ‘danger signs’, which can be identified by caregivers, and the application should facilitate the early reporting of these indicators to the immediate attending doctor. Alternatively, presence of these danger signs can be reported to another person or caregiver, who can immediately help the patient, for example, by accompanying him/her to the doctor. Other aspects of seeking treatment included the use of in-app checklists, such as what the patient specifically needs to do following clinical encounters. Clinicians also expressed interest in obtaining regular symptom reporting from patients via a mental health application.

##### 3.2.2.2 Sub-theme – medication side effects

Patients and caregivers provided suggestions on management of medication side effects, stating that the complete adverse effect profile of a medication could be informed through the application. Patients expressed the need to differentiate a clinical symptom of the illness from a manifested medication adverse effect. Among clinicians, they highlighted the importance of managing side-effects of ongoing medication use where mental health applications could allow patients to report their treatment experiences. Clinicians added that advice on management of different side effects could be provided *via* the mobile application. Clinicians also mentioned that patients could be given reminders through the application on bringing packets of consumed drugs during follow-up visits for the doctor to understand the level of medicine intake, which could be combined with blood tests to make informed decisions about the presence or likelihood of an adverse effect.

##### 3.2.2.3 Sub-theme – promotion of mental health knowledge and wellbeing

Patients and caregivers opined that any planned mental health application should have activities that improve the user’s overall mental or emotional state. Participants stressed the inclusion of tips or content in the application to strengthen a daily ‘mental health routine’, as well as capacities of an application to help beyond the management of clinical signs and symptoms and redressal of clinical queries. Participants highlighted the usage of applications to disseminate the right kind of mental health knowledge, particularly for rural users, and reduce superstitions around mental health conditions to promote care seeking. Caregivers also commented that mental health applications can play a role in improving awareness/knowledge of mental health conditions and their treatments.

Clinicians concurred with patients’ perspective that the app can serve the function of knowing more about the patient’s overall mental health, or making inferences on patient’s mental health by obtaining general data on routine functioning. Clinicians expressed this view by focusing on granular aspects of the patient’s daily schedule of activities. Clinicians also agreed that mental health applications should have a form of assessment of patient’s physical/fitness activities, and added that the app can provide guidance on activities to engage the patient, given the nature of his/her clinical condition, as well as mindfulness activities. Clinicians also identified that caregivers may need communication channels through a mental health application, such as closed virtual group meetings among caregivers to voice concerns, particularly in view of the larger social stigma surrounding mental health disorders.

##### 3.2.2.4 Sub-theme – Help-seeking and availability of medical help

Patients and caregivers noted several challenges in terms of availability of doctors to enable them to make the right choices. The difficulty in reaching a doctor during the lockdown situation in the wake of the pandemic was also highlighted. On being asked about the availability of a nearby family physician, a patient commented on the distance being “50 ​km*”* and highlighted that the treatment should be over the phone (Patient, pFGD-3). Caregivers also emphasized their interest in video consultations through an application. Clinicians stressed upon the need for an application to provide useable data on availability of medicines, access to financial support for treatment as provided by the government or other forms of financial aid such as concessions for medication costs.

#### 3.2.3 Theme 3 – How can caregivers be involved in the use of a mental health application?

Caregivers concurred with patients’ perspectives on mental health application usage, although, interestingly, clinicians primarily described the specific ways to support caregiver involvement. Clinicians expressed that they should be able to communicate with the caregiver through a mental health application in a structured manner as they perceived the caregiver to be a point person for transfer of knowledge. Clinicians also expressed that the caregivers can serve an important role of verifying the information that patients enter in surveys or symptom inquiry forms or check-lists developed within the application. Therefore, while clinicians wanted the patient-users to self-report mental health concerns, clinicians also wanted caregivers to review the situation independently at their level. Clinicians also suggested that the manual data entered in the application can be monitored by caregivers, or there could be caregiver-guided patients’ responses to symptom-related questions. In one instance, a clinician suggested for the caregiver to refill the same symptom survey, so as to *“collect the correct information”* (cFGD-2). Finally, a clinician also suggested for greater caregiver control *(‘user role’*) to increase the veracity of information reported through the application. Clinicians also recommended that caregivers’ be actively involved in the development of mental health applications.

#### 3.2.4 Theme 4 – How can a mental health application support doctors’ clinical work?

Clinicians suggested that a mental health application can have intelligence to store the reported data such as patient’s history, symptoms or treatment details of previous visits, and any improvement in the patient’s condition over successive visits, for usage in subsequent visits. Clinicians also suggested the need to reduce the effort of recalling previous visit data (in view of managing several patients), while also suggesting that an application can show a measure of change in the patient’s condition. Clinicians emphasized that an application can reduce the cumbersome process of logging patient’s history; however, they expressed scepticism on the ability of an application to capture and store the variations in a patient’s mental health history, particularly when it comes to patients with complex psychiatric disorders.

Clinicians also highlighted that the app should facilitate communication between doctors and patients through a question-and-answer system that is easy to follow for the patient. To identify patients' symptoms, the app needs to communicate in simple phrases that can be easily identified by patients without even asking or seeing the clinician - the latter was perceived to be important given the workloads of clinicians. These phrases or messages can be used by clinicians to better organize the symptoms and help prioritise patients, which in turn, will help in their continued follow-up with the clinician through the application. In addition, clinicians mentioned the separate presence of apps for scanning documents and the “sharing apps” for videos and photos (e.g., of clinical reports) but they seemed to indicate that a single app with these functions can make it easier for conducting their routine work.

With respect to clinicians’ perspectives on using applications to support caregivers, we found that beyond the clinic visits at the aforesaid Government hospital, clinicians are not communicating with caregivers via other channels, which could be partly due to the Government health system constraints (refer table-A.2, theme-4). Clinicians indicated that while they would want to communicate with caregivers through an app, there will be restrictions in the public health care system, and private practitioners will have more freedom to interact with caregivers *via* apps – for example, they could recommend apps to clients, track the usage of an app and communicate with clients on various topics through the recommended app. Yet, clinicians endorsed the use of mental health applications to provide direct support to caregivers, accentuated during the pandemic when caregiving at home became crucial. Clinicians added that technology can play a key part to bridge the access gap in situations like these, or more generally, they suggested that an app can use content from some of their existing in-person/group interactions with caregivers. Clinicians stressed the use of a mental health application to reduce what they referred to as ‘caregiver burden’.

### 3.3 Inductive themes

The data obtained in FGDs also helped us to identify two inductive themes discussed below (Table-A.3).

#### 3.3.1 Theme 5: Qualities of a mental health application

Patients’ and caregivers’ offered reflections about features or functionality aspects of a mental health application that they desired. For instance, use of non-medical, simple and understandable app-language, translated into local language was repeatedly emphasized across all patient-caregiver discussions. Participants reinforced the value of simple app messaging, particularly for patients living with schizophrenia. There was also a preference noted for minimum user effort, where users desired information from an application directly, and without any conditions on the application or without making them take additional steps. The ability for an app to provide urgent and specific information in emergency situations was also deemed as important.

In one instance, a caregiver of older age, suggested that while he is not comfortable with using technology, he has found an application used by his son (i.e., the patient) on accessing medicines to be simple enough and got an idea of its usage. This suggests the possibility of shared usage of applications between patients and caregivers, especially in families with differential technological comfort levels among individuals. This also underpins the point of simplicity in app functioning, to the extent that caregivers of older generations can be engaged in the app to support the care for a loved one living with schizophrenia.

Similarly, among clinicians, there was emphasis on technological simplicity and the importance of language translations, particularly in India, where they strongly recommended a mental health application to be multi-lingual and not *“too technical”*; for example, not involving too many steps, or heavy use of language, or complex terms in its interface.

#### 3.3.2 Theme 6: Data privacy and confidentiality

Patients and caregivers indicated situations where they could be comfortable in sharing personal data and instances where they would not share personal data. There was a certain degree of acceptance among participants of the fact that some amount of data sharing would be necessary if they needed to benefit from the strengths or services offered by a mental health application. Participants’ initial responses revealed that they seemed comfortable with location data sharing in a fitness app, given the purpose of this app to track step count or distance covered, as well as for purposes related to seeking treatment or accessing a care facility. One participant mentioned that if an application needs to offer personalized suggestions to the user on any aspect such as treatment or care-seeking, it would need to *“track our historical data”.* However, at the same time, participants were conscious of breaches in data security and privacy, and encouraged the use of permissions to access personal data depending on the situation, and not a one-time direct access of data. Clinicians largely agreed with the above aspects and provided a broad position on protecting the confidentiality of patient data.

## 4 Discussion

Few qualitative studies in India have examined the perspectives of individuals living with schizophrenia, their caregivers and clinicians in adopting and using mental health applications to improve care-seeking, illness management and remote care. Our study offers novel insights about the perceived needs and barriers among these different participant groups in using mental health applications in a government funded public institution for provision of psychiatric care in Madhya Pradesh, India. Table A 4 offers a summary of these key findings.

This is one of the early qualitative studies in India examining the perspectives of key groups of potential users of a mental health application. To our knowledge, this is the first such study conducted in Madhya Pradesh, a lower resource setting where there exists a serious mental health burden and treatment gap. The scarce research on this subject has mainly focused on caregivers’ needs and barriers in the design of mental health applications, but not that of patients and clinicians, which are highly important user-groups (Sinha et al., 2018). Our exploration of patients’ and clinicians’ perspectives on the content and features of mental health applications, potential usage of applications to communicate with each other, ways to involve caregivers in the process of treatment, and for clinicians to improve the efficiency of their routine work is crucial to build the evidence base in India on digital mental health applications, which is an urgent mental health policy priority. Moreover, the research so far has explained broad caregiver perspectives (Sinha et al., 2018), for example, usage of apps to improve medication adherence, patient’s connectivity with doctors, tracking of symptoms, and promotion of mental health literacy. Our exploration into these themes offers specific suggestions on app functionality and components, which can guide the frameworks that would be used to develop digital mental health applications.

Among clinicians in this study, we found that they seemed to recognize that mental health applications could provide a new means of communicating with patients and caregivers and supporting remote care and oversight of patients’ symptoms or mental distress. Interestingly, even with a diverse age range among participating clinicians, there did not appear to be any preferences towards face-to-face contact for providing care, compared to an application. The clinicians seemed to recognize that the two treatment channels could be supportive of each other. This is an important observation and distinct from prior studies where only more digitally-savvy doctors would be more comfortable with apps (Bucci et al., 2019; Gagnon et al., 2015). This could partly be explained by the existing high workloads on doctors in traditional clinic-based care provision settings in India, as well as the rapidly emerging digital health field and high smartphone penetration across much of India.

Clinicians also did not express any lesser preference towards digital communication with patients compared to traditional clinic appointments, due to reasons found in other studies, such as absence of warmth, nuance and empathy in a therapeutic relationship (important for clinical outcomes in mental health) due to the digital format of communication (Berry et al., 2017; Bucci et al., 2019; Sinclair et al., 2013). This could be partly due to the low patient-clinician interaction time given high workload, which could have prompted clinicians to be more receptive to other channels of communication such as digital applications.

Specifically, we found that patients and caregivers stressed the use of mental health applications to not only alleviate symptoms or distress but also to promote mental health knowledge, and particularly in rural or hard-to-reach users, to ensure the correct knowledge to overcome superstitions, irrational beliefs, or misinformation around mental disorders. Prior studies have also viewed digital solutions as a form of ‘social equaliser’, or having a potential to improve social inclusivity *via* dissemination of a common knowledge base to all, including hard-to-reach groups (Berry et al., 2017; Robotham et al., 2016). One intriguing finding was that caregivers expressed wanting to use mental health applications for *“a little bit, before approaching a doctor”*. This suggests the prevailing stigma around care-seeking for mental disorders by visiting a formal health facility, as reflected in previous Indian studies (Kishore et al., 2011; Sinha et al., 2018). Yet, this also highlights the potential receptivity of users to obtain knowledge/psychoeducation on mental health conditions in the comfort of their private space (confines of an app) before they seek professional care.

Among all participant groups, there were clear recommendations for use of simple, non-medical terms as part of app language, and importantly, the need for a multi-lingual mental health application, which reflects the fact that although smartphone usage has expanded in India, use of regional or local languages in apps will be essential for ensuring comprehension across diverse settings. Indeed, only 10% of Indians use English as a second or third language (Ministry of Home Affairs., 2011) and ∼45 million active Internet users (including 64% rural users) access Internet in their regional language (Telecom Regulatory Author, 2016). Our findings on language are consistent with those found in recent Indian research on acceptability of digital mental health applications (Sinha et al., 2018), where acceptability is more related to the user’s comprehension of understandable and useable content, and not only to the user’s knowledge of a second or third language. Periodic pilot testing of an app for user feedback on language, therefore, becomes necessary given the breadth of mental health content and its sensitive nature, which again underscores the importance of a participatory approach of involving patients/caregivers and clinicians in development of apps.

Interestingly, clinicians indicated uncertainty regarding their position on using patient’s self-reported data for remote supervision and care collected through mobile apps – even though clinicians encouraged self-reporting, they also recommended caregivers to oversee and verify the self-reported data. This is concurrent with observations in other studies where input of data by patients could be prone to errors, such as incorrect dosage intake or false feedback about medicine intake to get rid of medication adherence, which could threaten the successful adoption of a mental health application (Joshi et al., 2014; Sinha et al., 2018).

While this study explored the doubts and anxieties of patients and caregivers on the inappropriate usage of their personal data entered in a mental health application, we did not probe the same fears among clinicians, as explored in prior studies (Chang et al., 2013; Gagnon et al., 2015; Irwin et al., 2012; Valaitis & O’Mara, 2005). This could be due to the fact that this was not one of our primary study objectives. However, research has pointed that data security concerns can act as barriers for adoption of digital mental health applications among clinicians as well, across a range of healthcare settings (Chang et al., 2013; Gagnon et al., 2015; Irwin et al., 2012; Valaitis & O’Mara, 2005), including qualitative research with mental health staff working with individuals with severe mental health problems (Berry et al., 2017).

Findings of studies involving mental health applications and digital capacity-building in India could be triangulated with the results of our study. For example, a study on the pilot evaluation of ‘POD Adventures’, an app-based guided problem-solving intervention for adolescent mental health delivered in Indian secondary schools with guidance by lay counsellors, integrates face-to-face contact with self-guided digital content and its approach is consistent with findings that human support can optimise engagement with digital interventions (Gonsalves et al., 2021). This concurs with themes of the present study where patients expressed the need to stay in touch with the clinician (follow-up visits) while providing data on their symptoms through the application (e.g., self-assessment, symptom reporting and feedback) and differentiating a medication side-effect with an illness symptom with the help of the attending clinician. Another study on digital training of rural frontline workers to deliver a brief psychological treatment for depression in India (Muke et al., 2020) revealed practical user-related challenges such as poor connectivity, smartphone app pages not loading, and difficulty in navigating the course content, which although in a rural setting, could reflect similar potential challenges in urban areas of low and middle-income countries. While service satisfaction scores were high in the aforesaid study with adolescents, the heterogeneity of skills in using digital applications across socio-demographic and cultural groups in India and the anticipated technological challenges as cited in the study with frontline workers, suggest a need for introducing interventions to increase digital/smartphone competence and independence of users having mental health conditions. Digital Opportunities for Outcomes in Recovery Services (DOORS) is a pragmatic hands-on group approach in the United States with a similar goal for individuals with serious mental illness (Hoffman et al., 2020). DOORS was implemented in two different settings: a first-episode psychosis program and a clubhouse for individuals with serious mental illness, which resulted in the production of publicly available, free training manuals to empower others to run such groups and adapt them according to local needs.

### 4.1 Limitations

Our qualitative study has certain shortcomings to consider. We have analysed data as a secondary step, based on a guide primarily used to obtain feedback on the ongoing development of a specific digital mental health application (section 2.1). Therefore, our scope to probe patients, doctors and caregivers on various themes was limited, for instance, the insufficient probing of doctors’ fears and anxieties on threats to their data privacy and confidentiality, relative to the probing of patients on the same. We also have an under-representation of caregiver-specific perspectives under the patient-caregiver FGDs, which is partly because a significant part of the discussion was also dedicated to the demonstration of the basic version of the mindLAMP application. Most studies in India have not adequately explored caregivers’ wellbeing and needs, while they facilitate recovery of patients and fewer interventions in India have addressed *‘caregiver burden’*, as quoted by one of our participants (Chakrabarti, 2016; Leggatt, 2002). We also realise that caregivers could have provided more perspective on the potential risks of patients using mental health applications, as cited in previous Indian studies, such as ‘overuse of a mobile phone’ under the excuse of using the app, and ‘internet addiction’ and ‘gaming abuse’, which may disrupt the maintenance of a daily routine as advised by the doctor (Sinha et al., 2018). In this respect, given the mixed group of patients and caregivers, we could not identify potential problems due to patients or caregivers not fully expressing themselves before each other. If this was a primary data collection design, we could have separated the patients and caregivers in different focus group discussions to potentially eliminate the effects of power dynamics and better facilitate the expression of emotions during focus groups.

Participants may have also faced peer pressure that could have influenced their thoughts or opinions during focus groups. Some of the study procedures aimed to minimize these, for example, during the screening of patients (outpatient clinic), the attending clinician oriented the participants to the study objectives and after a thorough discussion on their willingness to participate, the clinician offered them to contact the research team for a further orientation and consenting procedure (in a separate venue). Any concerns on study participation, usage of their data, and protection of the information to be provided in focus groups were discussed and resolved during this process, before formal consent was obtained. In addition, the participating patients have been visiting the clinician for several months or years, thereby establishing trust with their attending clinician, prior to their role in the study.

We have also realised that further research with clinicians, caregivers and patients is needed with tailored questions on their experiences of living with and treating schizophrenia, as the current study was not primarily designed to obtain unique perspectives of participants on schizophrenia care, both at home and *via* the remote clinician. Moreover, the study sample had a limited scope i.e., urban tertiary care-level providers, hospital patients and caregivers, which can challenge the generalizability of results to other settings in India such as primary or community health centres in rural areas or private healthcare facilities. This also applies to the education profile of our study participants, limiting the generalizability of findings to participants having a lower educational level. The study also could not directly measure the capacity of the three kinds of participants to use mental health applications. Finally, impact of COVID-19 on availing and accessing mental health care and clinicians’ concerns of reaching out to their patients may have influenced their overall viewpoints.

## 5 Conclusion

Our parent study (Rodriguez-Villa et al., 2021a, 2021b) is guiding the systematic development and adaptation of an open-source smartphone application called “mindLAMP” across diverse settings in Boston, Bengaluru and Bhopal, to promote personalized care, with the goal of predicting and preventing relapse among individuals diagnosed with schizophrenia spectrum disorders. Findings of a qualitative study integrating the perspectives of patients, caregivers and clinicians obtained through focus group discussions across the three sites, specifically on mindLAMP, with respect to its features, functionality and utility have been recently published (Rodriguez-Villa et al., 2021b). Findings of the present analysis that focus on user perspectives towards mental health applications in general, indicate themes that are largely similar to the aforementioned paper on mindLAMP and build on the observations of the former manuscript (Rodriguez-Villa et al., 2021b) such as participants being open to integrating technology into treatment to better understand their mental conditions and inform their treatments; recognizing the need to report symptoms, medication side effects and emergency concerns to mental health professionals on a dynamic basis (particularly after the pandemic); emphasizing the need for an accessible user interface and app language; and sharing concerns on the usage of their personal data. Findings from both the manuscripts have been communicated to the software development team, which is working on refining the mindLAMP application (BIDMC, Boston) and have been duly considered by teams of the Indian sites to incorporate customized features into the mindLAMP application for usage in individual settings, as part of the upcoming phases of the parent study (Rodriguez-Villa et al., 2021a).

Specifically, findings of this qualitative analysis represent a step forward in building the much-needed evidence base in India on perceptions around digital mental health applications, and their desired components, ways of usage, and barriers to adoption. These findings will be valuable for informing our subsequent work, but also offer important insights for broader research efforts aimed at developing digital mental health interventions for low-resource settings. In a country with a tremendous mental health treatment gap for individuals living with schizophrenia, the building of evidence on the strengths and challenges of using digital applications is essential to fulfil the specific needs of these patients, and of their caregivers, as well as their attending clinicians.

## Author contribution

**Ameya P. Bondre:** Investigation, Validation, Formal Analysis, Writing – Original draft preparation, Visualization. **Ritu Shrivastava:** Investigation, Data Curation, Validation, Formal Analysis. **Harikeerthan Raghuram:** Investigation, Validation, Formal Analysis. **Deepak Tugnawat:** Resources, Data Curation, Supervision, Project Administration, Writing – Review and Editing. **Azaz Khan:** Writing – Review and Editing, Supervision. **Snehil Gupta:** Writing – Review and Editing, Resources. **Mohit Kumar:** Resources, Project Administration. **Urvakhsh Meherwan Mehta:** Writing – Review and Editing, Supervision. **Matcheri Keshavan:** Writing – Review and Editing, Supervision. **Tanvi Lakhtakia:** Software, Resources, Project Administration. **Prabhat Kumar Chand:** Writing – Review and Editing, Supervision. **Jagadisha Thirthalli:** Writing – Review and Editing, Supervision. **Vikram Patel:** Conceptualization, Methodology, Supervision. **John Torous:** Conceptualization, Methodology, Software, Writing – Review and Editing, Funding acquisition. **Abhijit R. Rozatkar:** Writing – Review and Editing, Resources, Supervision. **John A. Naslund:** Conceptualization, Methodology, Writing - Review and Editing, Supervision. **Anant Bhan:** Conceptualization, Methodology, Writing - Review and Editing, Supervision, Project Administration.

## Funding

This work was supported by the 10.13039/100010269Wellcome Trust, UK (grant no. 215843/Z/19/Z).

## Data statement

Due to the sensitive nature of the questions asked in this study, the respondents of focus group discussions were assured that raw data would remain confidential and would not be shared.

## Declaration of interests

The authors declare that they have no known competing financial interests or personal relationships that could have appeared to influence the work reported in this paper.

## Figures and Tables

**Table A.1:** Participant demographic characteristics

FGD #	N (total = 25)	Age-range	Education level	Patient’s gender	Relationship of caregiver to patient	Range of # of years of receiving treatment
1	*Patients* = 4 *Caregivers* = 4	*Patients:* 23–42 years *Caregivers:* 23–73 years	*Patients:* Undergraduate: 0 Graduate: 3 Postgraduate:1 *Caregivers:* Undergraduate: 1 Graduate: 1 Postgraduate: 2	Males = 2 Females = 2	Mothers: 1 Fathers: 2 Siblings: 1	1 year–15 years
2	*Patients* = 4 *Caregivers* = 5	*Patients:* 18–41 years *Caregivers:* 18–52 years	*Patients:* Undergraduate: 3 Graduate: 1 Postgraduate:0 *Caregivers:* Undergraduate: 3 Graduate: 1 Postgraduate: 1	Males = 1 Females = 3	Mothers: 2 Fathers: 2 Spouse: 1	1 year–10 years
3	*Patients* = 3 *Caregivers* = 5	*Patients:* 19–32 years *Caregivers:* 20–61 years	*Patients:* Undergraduate: 0 Graduate: 1 Postgraduate:2 *Caregivers:* Undergraduate: 1 Graduate: 3 Postgraduate: 1	Males = 2 Females = 1	Mothers: 1 Fathers: 2 Siblings: 2	10 months–4 years

FGD #	N (total = 19)	Age-range	Gender	Designations
4	Clinicians = 7	32–45 years	Males: 6 Female: 1	Mental Health Educator/Social scientist: 1 Consultant Psychiatrist: 2 Medical Social Service Officer (Mental Health): 1 Senior Psychiatry Resident: 1 Senior Nursing Officer: 1 Child Psychologist: 1
5	Clinicians = 6	29–41 years	Males: 3 Females: 3	Medical Social Service Officer (Mental Health): 2 Senior Psychiatry Resident:1 Vocational Counsellor: 1 Psychiatry Nursing Officer: 1 Senior Psychiatry Nursing Officer: 1
6	Clinicians = 6	27–36 years	Males: 3 Females: 3	Senior Psychiatry Resident: 2 Assistant Professor Psychiatry: 1 PGDRP Clinical Psychology: 1 MPhil PhD Psychology: 1 MPhil PhD Psychiatry Social Work: 1

**Table A.2:** Deductive ‘a priori’ themes

Theme	Participant type	Codes	Quotes
***Theme 1: Prior experiences with health applications***	Patients and Caregivers	Emphasis on physical fitness	*“Yes, we count our steps, distance and even we can calculate weight gained or lost.” (pFGD-2)*
		Mental health gains out of physical fitness apps	*“It (the particular fitness app) gives me pleasure, and shows that these many kilometres have been walked and these many calories have been burnt. It gives me mental satisfaction that you have put in that much of physical effort.” (pFGD-1)*
		Comprehensive health information	*“(the app) shows brands (medicine brand names) of all companies and also how to use it (the medicine), what are the side effects, what is the usage like. You will find everything at one place in one app”. (pFGD-1)*
		Seeking help from qualified expert	*“Everything is there (in the app). Means, we have to fill patient information in that (app). Then, there are doctors and MD doctors, they tell everything about what to do next, what precautions you have to take …” (pFGD-1)*
	Clinicians	Simple features of a lifestyle modification app	*“Mostly it* was *easy only, since it included simple food items. And whatever you entered, like if you entered ‘chapati’ (bread), chapati was coming (in the search box). You enter roti (different kind of bread), roti was coming, or if you enter dal (lentil soup), etc. There were no English words. It was very simple! You could easily calculate calories. And it (the app) has a diabetes coach as well. And time to time, every 15 days, they (doctors, coaches) would call the patients.” (cFGD-2)*
		Coaching for lifestyle modification	*“This app has different meditation techniques. For a passive recipient, this becomes easy, somebody is guiding them.” (cFGD-2)*
		Lifestyle modification app for low-literate users	*"I was doing a lifestyle modification programme for metabolic issues, for my patients. So, I used this BMI app and I requested them to download it, so that they can frequently monitor their BMI. There were issues, such as patients were from very rural areas, and some were not having smartphones. So, for them it was difficult. But many of them could download it and use it properly, with the help of family members. I taught them manually that they can do it.” (cFGD-1)*
		Dispatch of medicines Exposure to a mental health app	*“There are medical pharmacists which are sending medicines through couriers. This is a new concept now. Net meds is a company which is sending medicines directly to the patients on prescription.” (cFGD-3)* *“Once, I had asked patients to use an app on cognitive retraining. However, I don’t recall their experiences with it.” (cFGD-1)*
		Communicating with mental health patients in pandemic	*“I communicated with my patients using WhatsApp video and WhatsApp chatting. Many of the patients could not come, they were from outside Bhopal” (cFGD-3)* *“I wish we had more apps to be able to communicate more easily with our patients because we normally prescribe our patients drugs for one month and in between if they have any complaint, they don’t really visit or, they are not able to come repeatedly to ask questions, so we should have a lot of (continuing) communication with our patients. So, an app will be better than the (patient’s) mobile ringing all the time”* *“In one instance, during pandemic, our department initiated a tele counselling facility.” (cFGD-3)* *“We got phone calls and they were all for medicines or non-availability of medicines during pandemic. So, I think the doctors should see to it as to how the medicines reach the patient in such times.” (cFGD-3)*
		Apps for research purposes	*“Actually, this is a mobile application for reducing weight. It was used in a study where two groups were made. One group of patients were to be given just the app. One group were to be sent to dieticians. And, we were to see the difference between how each group reduced their weights.” (cFGD-2)*
***Theme 2: What should a mental health application have? – Sub-theme: Signs and Symptoms***	Patients and Caregivers	Self-assessment and reporting; addressing queries; questionnaire flow for the same	*“If there is something where we can put our symptoms and take some advice from the app, or what should be done for relieving these symptoms. What should I do in this kind of a situation and how can I be healthy? Or what precautions I should be taking? All these things should be included in (such an) app.” (pFGD-2)*
			*“If a user is ill, then there should be an option in the app to elaborate the nature of the difficulty, like it’s there in Aarogya Setu (Government application for tracking of COVID-19 symptoms). But, in it (Arogya Setu), the detailing on symptoms is not much, so this app (mental health app) should ask more details. There should be a system, where if you keep clicking, then new questions will appear and you would put more details on symptoms, to finally arrive at a solution.” (pFGD-1)*
			*“So, there should be options. For example, if I have an illness, then the app should automatically ask me, whether my illness is of this type or that type (categories or drop-downs), so that I can find it easy to click and go ahead and there will be an emergence of a solution.” (pFGD-1)*
		Need to communicate symptoms of an urgent nature	*“If there can be a ‘panic button’, so that when needed, it can be pressed in the app. And via Bluetooth or internet, the message should reach the doctor (or concerned medical contact). Without (an app requiring) internet, this will not be possible. This feature should let us raise an alarm so that we receive medical intervention in the minimum possible time.” (pFGD-1)*
			*“Like our family members, say 5–6 of us, we should come to know (of the alarm). And, plus the doctor. So that they (family members) get the feedback from the patient immediately and can immediately help the patient (or arrange referral).” (pFGD-1)*
			*“Sometimes it so happens that this (anxious) person cannot do anything, he doesn’t understand what is happening. This will help then.” (pFGD-1)*
	Clinicians	Psychoeducation on symptoms	*“So many patients get relapse of the illness because of not of understanding about the disorder. So, they quit the medication, they quit the therapy and they are having recurrence of the disease and they are again in the same situation”. (cFGD3)*
			*“First of all, if we target our population in our country, illness awareness is a big thing. It should be done (in app). We can go with the model of psychoeducation” (cFGD3)*
			*“If someone is suffering from schizophrenia so we need to tell (him/her) what is schizophrenia, how it happens, what is the course of illness, how do I treat, what are the chances of relapse and what is the chances of recovery, all these things, if we are talking about an app then this information should be fully given” (cFGD3)*
		Emphasis on specific symptoms or ‘target’ symptoms	*“Recognizing symptoms (in an app) is fine… but what is the target? That should be very specific. You know "target symptoms" in the patient? They must be there in the app.” (cFGD-1)*
			*“What are the symptoms, to which probably the patient is showing improvement, he should know that in these areas he is getting the improvement” (cFGD3)*
		Reporting of key signs and symptoms; Use of a checklist to arrive at a decision	*“Like in schizophrenia or sometimes in mood disorders, there are signs that can be spotted early. Then they (patients, caregivers) should immediately go to their attendant doctor, or the signal should go to another close person, that they should attend the patient." (cFGD-1)*
			*“When we look at an application like this, like, to find out the stress level of the person, or whether the person is having a suicidal thought, or depression…. so, we need to go through some checklist, which the patient can fill and submit. Then, he will get one thing that - whether he should get this (particular) kind of treatment or something else. He should get some kind of notification that 'you need to get consulted with the doctor'.” (cFGD-1)*
		Knowing granular details of a patient’s symptom	*“I would want to know the mood of the patient. How happy he is; or side effects of a medication or what is the status of the medication that they are taking regularly. I want to know what they are feeling. They can rate the mood from zero to ten or like that…And if it is live, then at the right time, they can fill the right data through the app.” (cFGD-2)*
***Theme 2: What should a mental health application have? – Sub-theme: Medication side effects***	Patients and Caregivers	Differentiation of symptoms due to illness and medication side effect	*“First of all, whatever medicine we are taking … what will be its reaction or what will be the side-effect – that information we should get in advance of starting the medicine. And in detail, because if using a certain medicine would lead to a certain problem, then we should know that if tomorrow that problem occurs, then it is due to the medicine. So that, we are not mentally stressed.” (pFGD-1).*
			*“What would be the effect of the treatment on the symptoms? That would be something that a person would like to be aware of. If not for the patient, then the relatives can know about those things. And they can prepare themselves for that. That too, not too much information, just basic information must be there.*” (*p*FGD-3).
	Clinicians	Managing side effects as they emerge	*“For mental health assessment, the app should be very specific for a disorder, so that it will be good for the patient, like, suppose somebody is diagnosed to have bipolar affective disorder or schizophrenia, then the app should categorise the symptoms and make it very specific to the patient – be it warning signs or side effects of medication.* *Mostly patients under treatment will discontinue medication because of side effects. It might be due to a simple weight gain and patient will just stop the medication. So, if they are thinking of stopping medications, then what can they do after that? There should be some facility in the app like, they can send a notification to their caregiver or doctor that they are having this problem and they need a consultation. So, this kind of notification will definitely help”. (cFGD-1)*
			*“The person using the app should have a list of side effects; so, whatever he or she faces, there should be (in-app) tips on managing that. This will also help us to see a record of side effects faced by the specific person.” (cFGD-1)*
		Suggestions to reduce side effects (in-app)	*“This kind of app should have an alarm or something so that the patients can be particular about the times at which medicines have to be taken.” (cFGD-1)*
		Reminders to bring medicines to take decisions on testing for side effects	*“We can add reminders for them, it can be reminders for various medicines, because when they come for follow up, they say we have forgot the medicines. So, we cannot do routine blood investigations in cases of many drugs (these are done to assess blood levels of medicines).” (cFGD-3)*
***Theme 2: What should a mental health application have? – Sub-theme: Promote mental health knowledge and wellbeing***	Patients and Caregivers	Activities to improve mental state; Strengthen daily ‘mental health routine’	*“There could be some light-hearted activities in such an application, where if we feel a bit anxious or something like that… then we can go to the application and have a look at it. There could be light-hearted quizzes; I mean, my attention and concentration should be diverted from my anxious thought and I should feel relaxed at that time.” (pFGD-1)*
			*“The app should give suggestions for conducting activities in daily routine and how to balance the mind through these activities, and how to keep mind focused, or on track …”* (*p*FGD-2)
		Right kind of knowledge/tackle stigma	*“Yes, there should be mental health apps, for people like us who are literate, who understand about mental health. But, in small villages, where people are uneducated, they usually look at mental health disorders by (from the lens of) superstitions. Therefore, it is necessary that they should not get distracted… and rather, come to places which provide mental health care facilities.” (pFGD-2)*
			*“Some people believe that hearing voices in our head (in the context of schizophrenia patients) or having mental health related symptoms is something unnatural and they connect it to superstitious things such as spirits, or unholy things and therefore, seek religious help. But, if the right awareness is available through mental health applications, then it would be the best.” (pFGD-2)*
			*“City folks understand about this. But in villages, the ignorant folk, whatever happens, they go to faith/spiritual healers or go to godman healers. They should also become aware that mental health problems are also a disease, just like physical diseases. Mental diseases can also happen, and there are doctors for that. This can be treated.” (Caregiver, pFGD3)*
			*“That this (mental health related problem) is curable with medicines. Many [people] think that the [medicines] have to be taken life-long, which may happen in rare cases. Or they think that it will be become like a habit, an addiction, that people cannot function without it. Like that, many things come into our mind.” (Caregiver, pFGD3)*
			*“I want to say this thing that, we should not reach the wrong place, and think of traditional beliefs, for example, that the mental illness is because of a ghost. There should be some awareness generation as well the fact that all these [mental illnesses] are also medical diseases.” (Caregiver, pFGD3)*
	Clinicians	Details of routine patient functioning	*“I want to know - How long do they study? When do they eat and for how long? And also, about sleep… How long do they sleep…? I want to know through the app. When do they go to sleep and when do they wake up? Do they wake up in the middle of the night? Through the app we can ask them to fill this [information]. The scheduling of their activities is needed. What time a person is doing what? For instance, at night time – what are his activities, and today what time has he gone to bed …” (cFGD2)*
			*“At the end of the day, we (want to) know what our patient had done, the activities of the whole week; that is very useful for us to know. The caregivers can help provide this information” (cFGD3)*
		In-app activities to engage the patient	*“So, what I am imagining is a mental health app where there are activities for ‘initial phases’ of conditions, for initial phases of anxiety or something like that. We can list out those activities for the patient, we can organize these activities so that it is very easy for the patient (to follow) and we can use different links and different languages.” (cGFD-3)*
			*“I think it should be for physical and mental health. Physically as [participant name] Ma’am earlier said, there should be meditation (activities in app), and, mentally, I think there should be, if there is any option for cognitive retraining, or for memory, or concentration and if there is any option for yoga, then I think that would be very helpful for patients.” (cFGD-3)*
		Group interactions for caregivers through the app (tackle stigma)	*“Because schizophrenia patients are in not that condition, that they can convey (issues) to their family members and this is a very difficult situation for the caregiver; also, if there is any option for family members to talk with other family members. I mean patient has a point of view but caregiver is also in a very difficult situation. If there any option for a group meeting, I think it may be helpful.” (cFGD3)*
			*“There is always stigma and in relapse, stigma plays a very great role. So, if they (families) are getting social support (in-app), if they are coming up as a group and if this app helps them to connect to other caregivers with a similar sort of diagnosis, then I guess it will be a great initiative.” (cFGD3)*
***Theme 2: What should a mental health application have? – Sub-theme: Help-seeking and availability of medical help***	Patients and Caregivers	Decision making on where to avail medical help	*“Now I felt that when I am in a situation then where to go [for medical help], which doctor to visit, who is appropriate for this [problem], for the symptoms that I have, for that, is there one doctor or 15 doctors. And among them who is nearby to me, which is the right place where we can start treatment, where the mother, father, or whoever is the relative, they can easily take them there. If there is something like that in the app, then time won’t get wasted, and we can get treatment [which is most appropriate] for the symptoms” (Caregiver, pFGD-3)*
			*“In the primary stage [our worry is] how we will reach the right place, which doctor should we go to, whether the doctor will be authentic or not. These things trouble us a lot in the primary stage. It hits us regarding where will we go. At that time [we get suggestions] from somewhere, someone, we get in touch with someone, with friends, then they tell us, they tell us something based on their experience. They might have a relative somewhere or the other, or they may know someone like that, so they tell us.” (Caregiver, pFGD-3)*
		Difficulty in lockdown situation	*On being asked about the availability of a nearby family physician, a patient replied:* *“*50 km*. So that’s why I felt that [the treatment] should be through phone.” (pFGD-3)*
		Need of video consultations through the app	*“I think that if it is an app, then video calling and video conferencing can be done with anyone.” (Caregiver, pFGD-3)*
			*“However, doctors don’t have their numbers [put up], and it is not possible to contact the doctors. So, what happens is that we just search on the phone and whichever doctor is nearby or whoever is treating us, we will continue to get treatment from them. If they can’t come, then those doctors through the same app, by creating an ID, a link can be sent and they can come to us through video conferencing. Talking to them is one thing and seeing them in person is another. So, video calling should be there in the app, so we can speak with the doctor or the doctor can tell what medicines to take.” (Caregiver, pFGD-3)*
	Clinicians	Information on mapping of chemists for medication data	*“There is a mapping of chemists who are there, and where psychiatric medicines are available and this mapping should be available to both the patient and the treating doctor. So, in case of emergencies, those chemists can take the responsibility to make the medicine available to the patient. I don’t know how this mapping can be possible, listing of this and how we can bring those chemists and pharmacists on board in our app”. (cFGD-3)*
		Information on financial aid and Government schemes	*“So, links to proper agencies like government agencies where the patient can communicate, in case of whatever (treatment) benefits (or subsidies) are given or what services can be provided by government agencies. So, those links should be available to caregivers through the app.” (cFGD-3)*
			*“If this app can help us, all of us, to make them aware of any financial aid or any aids which they can avail. We ourselves as professionals are not aware at times. Because it is absent in our training part – but not in case of psychiatric social workers or psychologists. So, we don’t know where we can refer our patient where they can get financial aid. But we know there are some (government) schemes.” (cFGD-3)*
			*“Once they are diagnosed, then they may get some concessions, like for medicines that are of high cost. So, this app can provide us this kind of information also.” (cFGD-3)*
**Theme 3: How can caregivers involve in the use of a mental health application?**	Clinicians	Structured communication between caregivers and doctors	*“If caregivers are provided with a mental health app, then they can definitely be an asset for the patient’s recovery. So, day-to-day monitoring of the patient, for example, and I think the caregivers should be taken into account before providing apps.” (cFGD-3)*
			*“The family members of psychiatric patients have gone through a lot. All of the things that we are mainly communicating with patients, such as medications, diet, etc., we communicate through family members only. For the maximum time, patients are not able, or not ready to listen to us (our advice), or not ready to take the medication from us. So, we are giving supervision and conveying most of the things to the family members.” (cFGD-2)*
		Review/Monitoring of data entered by patients in the app	*“When patient answers this thing (symptom questions), the caregivers should be at home or caregivers should tell (verify) that they (patients) are taking medications; or that this (the survey) is done by the patient and the family member.” (cFGD-2)*
			In one instance, a clinician suggested for the caregiver to refill the same symptom survey, so as to 'collect the correct information.'
			*“Caregivers can also be given a user role in the app, to access their patient’s data. So, there is the patient [role], the clinician [role] and this (caregiver role).” (cFGD-2)*
		Caregivers’ role in development of the app	*“I think the app that is being developed should definitely have a say from caregivers and - how friendly they think it (the app) is for patients, and how can they (caregivers) contribute to this engagement with patients. Are there things which caregivers specifically want to be included in the app? So, definitely it should have a participation from caregivers as they are the users of this app.” (cFGD-1)*
**Theme 4: How can a mental health application support doctors’ clinical work?**	Clinicians	In-app intelligence to store and use data reported by patient (history taking)	*“So, when we are documenting patient’s condition, generally, we mostly write in scientific terms. So, it is something for us to understand. If the patient is following up, we know that these were the symptoms (he/she had) earlier, and how gradually with the treatment whether these symptoms are resolving or not. Now, what would help us is that if we see certain options, which tell us what we have chosen last time, or the symptoms that patient was having in the last visit, or this was the treatment which we had given, or what did the patient have (symptom) at the earlier time and how much it changed due to medication.” (cFGD-1)*
		Challenges in capturing patient’s history and what an app can do	*“History collection is the big work in psychiatry I think, so yes, the application will sometimes be helpful for collecting patient’s history. But in apps, I don’t know how history collection will be exactly done …” (cFGD-2)*
			*“Day by day, they (patients) are changing the (reporting of) symptoms. One relative (caregiver) will come and tell one thing (during clinic visits). Another relative will come and tell another thing. Patient will tell a different thing altogether. So, this is a big work for an app to do.” (cFGD-2)*
			*“When we have a lot of patients, we have to use other staff members to log the histories, so an app can help, in which the patient or the relative or the caregiver can initially fill in (at least) the demographic details or initial history. This will save clinicians’ time.” (cFGD-3)*
		Facilitate communication with patient in ‘patient’s language’	*“So, suppose the patient has to communicate with us – if, certain very scientific terms come in the app, then the patient won’t understand. So, there should be simple phrases. For depression, for example, it could be - Do you feel sad all day? Or, for schizophrenia - do you have doubts too much, do you feel suspicion? These kinds of things, framed in absolutely single targeted lines can be picked up by the patient, without even asking or seeing us. Here we can see what problems the patient could have, and how we can keep them on a priority basis. Because, it is possible that there are too many patients with a doctor. So, we can prioritise. One thing is that, the app should do the work of [effective] communication, which the patient can easily pick up and we (doctors) can categorise that information (for further use).” (cFGD-1)*
		Use of functions to facilitate operational work	*“Actually, my problem is with the scanner. I use these things (scanners) but because of my workload, I find it stressful. I have to go to this thing (scanner) so many times for my work. And when it affects my work, I become stressful. And, I think there are (existing) apps that allow sharing of videos, photos, documents, PDFs etc.” (cFGD-2)*
		Context for communication with patients/caregivers in the setting of a Government hospital	*“We may or may not have their contact numbers and not specifically in case of every patient. But if there is a high risk of relapse or if the patient has taken early hospital discharge (referred to as ‘discharge against medical advice’), then at that point of time only, I will give my phone number to the caregivers, and will communicate if any problems arise (or when the caregivers reach out in case of issues) or by WhatsApp.” (cFGD-1)*
			*“General communication is only when they (caregivers) accompany the patient in the OPD or in the ward (for in-patients). Otherwise, it is through a mobile, if you have time for caregivers, because they will call you anytime and ask about it (the patient’s condition).” (cFGD-3)*
			*“I think there is one disclaimer, it’s that we (doctors in FGD) are all from the government setting, so we don’t have that freedom and that copyright of seeing the patient the way we want, or prescribing the patient the way we want, right? So, there are definitely some restrictions as per government policy. But many of these things will apply to a person who is doing private (clinical) practice, for example, how they want to interact with their clients and caregivers, at what time, at what ease …” (cFGD-1)*
		Supporting caregivers/communicating with caregivers/reducing caregiver burden/providing informational material for caregivers	*“The caregivers’ role is very important and communication with the caregiver can definitely increase with an app, because at the end of the day we can devote some time to check what the caregiver is telling us and respond to them”. (cFGD-3)*
			*“Definitely, if we have an app, and in that app, we can provide certain educational material or videos, which caregivers can read or watch, then it will be helpful for the patient as well as their caregiver. The caregivers will be knowing what to do when any relapse is happening, or if patient is stopping medications, then what next steps they need to take and the ways in which relapse can be prevented.” (cFGD-1)*
			*“We have group meeting with caregivers and all us staff members like psychiatric social worker and the psychologist communicate with them and like I said, this is not only need based but we also give them information booklets. We don’t have apps as yet for this, but we do give them booklets describing the illness, what are the needs of the patient and now we have started with videos also. So, those are other ways that we have to communicate with caregivers, where an app can come in …” (cFGD-3)*
			*“The app should have things that I can suggest for caregivers, means, there is the concept called as ‘caregiver burden’. So, I want to monitor if a person is having more caregiver burden, or I can suggest some management tips and things to prevent the burden, or suggest the (caregivers) to follow a plan …” (cFGD-2)*

**Table A.3:** Inductive themes

Theme	Participant type	Codes	Quotes
**Theme 5: Qualities of a mental health application**	Patients and caregivers	Simplicity in app functioning; Reinforcement of app simplicity due to the possibility of shared app usage between family members depending upon their individual app fluency	*“Suppose I want to know more about Schizophrenia, and I reach a button (in the app), which I click and do something as directed, then I get some information, etc. - but it should be very precise, not medical. Because the common person who will use it is mostly a non-medico. So, he should be able to understand in simple language. Though schizophrenia itself is a very heavy word [laughs], but, yet, the app should give simple definitive words. And, not very verbose. In trying to explain certain things, we use so many words and so many sentences! So, it should be to the point and precise. But the clarity should not be compromised. Brevity should not be at the cost of clarity.” (pFGD-1)*
			*“So, I personally do not use any app, but my son does everything of that sort – i.e., getting the details of medicines, their availability and what medicine, or ingredients etc., and I understand from there …” (pFGD-1)*
		Minimum user effort	*“That is the most convenient way - any app that is not asking me to do something, and it is supplying all the information on a click; so that’s the first thing. It may be an ideal one, may not be existing that way (among current apps), but at the same time, whatever we want to know, we should reach that information without any turn and twist.” (pFGD-1)*
			*“In Aarogya Setu (COVID app), we can enter symptoms, but it requires Bluetooth; so, if we don’t turn it on, the symptoms will not be known to anyone. This is a problem and this kind of requirement should be minimum (need of Bluetooth).” (pFGD-1)*
		Urgent information in emergency situation	*“Like, as in some emergency situation, we can know the name of the tablet we need, or we can know the name of available medicines for our condition, at that time. Example for Schizophrenia - there is Risperidol (brand name of medicine), but that is not available everywhere, at all times. So, in some way we can know the availability of commonly used medicines (brands).” (pFGD-1)*
	Clinicians	App in multiple languages	*“It should be available in different languages; if it is in different languages, it will also be culturally appropriate. For example, if the app is available in Bengali language or in Hindi. So, different state people… because in India we grasp quickly with our own mother tongue so it will be very easy to grasp, keep in memory and follow.” (cFGD-3)*
**Theme 6: Data Privacy and Confidentiality**	Patients and caregivers	Personal data for constructive or relevant purpose	*“No, that is not a challenge. That is fine. Let it know (our location data). In fact, for us, everyday it becomes a challenge that … we need to know, yesterday how far we walked and the details of it, and so, how much we have to walk today (if there is no app). As a constant reminder, the app sends a pop up (message) in the morning that today you have to burn so many calories. So, it needs to give you a regular reminder.” (pFGD-1)*
			*“Suppose you add all your information in that (app). And then it gives you automatic suggestions – for example, it can tell the nearest place to visit, or to visit someone, and we can find the address from it. And, if this is a rural patient, then it can guide him on how to tackle the situation.” (pFGD-1)* *One participant summarized this point by quoting that if an application needs to offer personalized suggestions to the user on any aspect such as treatment or care-seeking, it would need to “track our historical data”. (pFGD-1)*
			*“The app should synchronize data from nearest proximity. Suppose, if we are taking treatment from here (AIIMS Bhopal) but we are located far from Bhopal, then in case of any emergency situation, we should be able to find something in that location itself.” (pFGD-1)*
		Consciousness of privacy breaches	*“An app should not disclose/breach some information like (from) contacts, photos, gallery… that’s all.” (pFGD-1)*
			*“In contacts, there should be an option given to share our contact information with only five close relatives. Some option should be there, where we can tick mark the five people. Rather than tell everyone in the world, it will be a better option that the app can send messages to our five relatives on whom we are dependent. There is no concern on this.” (pFGD-1)*
			*“If something needs to be uploaded from the gallery like some photos or a medical prescription (report)… so there should be an option where we can upload these things from (the gallery). Not that the app gets access through your entire gallery …” (pFGD-1)*
			*“There should not be anything like automatic (data) access from the app end. An app should ask users (permission) before taking access. The app should be dependent on users to allow or not to allow the access.” (pFGD-1)*
	Clinicians	Broad position on confidentiality	*“Whereas in psychiatry, what we really need is confidentiality. So, an app which can help keep records confidential would be very useful. We can’t allow our patients’ records to be accessed by even other physicians without permission. So, any app used for this must promise confidentiality, which I think is a very important issue.” (cFGD-3)*

**Table A.4:** Summary of main findings:

Perceived needs		
	Patients-caregivers	Clinicians
Current exposure	Patients and caregivers used digital health applications largely for lifestyle modification purposes.	Clinicians looked at health applications as tools for specific purposes such as diet applications or those monitoring body mass indices with user-friendly interfaces, or applications that enabled guided meditation techniques for beginners, or apps used for research purposes.
Perspectives on content of a mental health application	Preference for an app asking details on symptoms in the form of a flow of questions, which probes the nature of symptoms to arrive at a ‘solution’ or steps that they can take, as also an application that can raise an alarm in emergency situations (e.g., the ‘panic button’ feature).	Expected an app to inquire into a patient’s ‘target symptoms’, particularly in schizophrenia, as also warning signs and symptoms that need more urgent attention than others, given the workload on clinicians; Also interested to know the granular details of patient’s mood and feelings through the application
Medication side effects	They wanted to know through an app, if an adverse symptom is due to the illness or the medication	Expressed an interest in knowing the details of how medications were exactly consumed (e.g., dosage times, any stoppage of medication intake), nature of side effects, and their timely reporting through the app
Mental health promotion	Expected an application to guide them on maintaining a daily routine of activities to keep mental balance and provide the right information to reduce irrational beliefs and superstitions around mental disorders.	Curious about the specific details of patients’ daily activities (e.g., sleep and activity habits) to understand the possible effects on mental health; Regarded the caregivers as key point persons for transfer of mental health knowledge *via* an application, and encouraged caregivers to verify the information filled in by patients in the app.
Improvement in clinical working conditions	–	Found app utility, for instance, with reference to logging of patient history across previous visits to save time in documentation and using app intelligence to achieve the same; technological support in clinical operations such as sharing of reports, images or videos; enhancing direct doctor-patient communication on the status of symptoms through a question-answer format in easy ‘patient’s language’; and providing educational material to caregivers to reduce their burden in managing the patient’s illness.
Data Privacy	Expressed a general agreement on the use of their personal data, if the purpose of using that data was clarified by the application, for instance, if the app used personal data to offer customized suggestions to seek mental health care; and due permission was taken before accessing their private information.	–
General application qualities	All participant groups agreed that the application should have a language that is ‘non-medical’ and easy to follow for the patient to maximise its usage (besides translation into a local language), with a simple interface that does not demand user effort and directly provides the information that is sought by the user, particularly the specific information needed in emergency situations (e.g., in case of medicines), and the application should not put additional steps or conditions for users to obtain the desired information. We also learned that these qualities are important considering the shared use of such applications between patients and their older caregivers who may have lesser technological or smartphone know-how.	
**Perceived barriers**		
	Patients-caregivers	Clinicians
	1. Emphasized a threat to data privacy if personal data was directly accessed from contacts, photos or other sections of their phones and/or shared with third-parties without due permission.	1. Do not seem to have complete trust in patients’ self-reporting and need caregiver verification, even though they encouraged patients to report symptoms 2. Perceive that capturing variation in routine patient history/symptoms, which is important in psychiatric care, maybe challenging through an application 3. Their direct interaction with caregivers *via* an app, could be limited due to government hospital constraints although they deem caregiver engagement to be very important.
	All participant groups discouraged the use of heavy, medical or technical language to facilitate app-based interaction between the patient/caregiver and doctor, which may reduce the end-user’s involvement in the application.	
